# Terrestrial arthropods broadly possess endogenous phytohormones auxin and cytokinins

**DOI:** 10.1038/s41598-022-08558-6

**Published:** 2022-03-19

**Authors:** Makoto Tokuda, Yoshihito Suzuki, Shohei Fujita, Hiroki Matsuda, Shuhei Adachi-Fukunaga, Ayman Khamis Elsayed

**Affiliations:** 1 Department of Biological Resource Science, Faculty of Agriculture, Saga University, Saga, 840-8502 Japan; 2 The United Graduate School of Agricultural Sciences, Kagoshima University, Kagoshima, 890-0065 Japan; 3 Department of Food and Life Sciences, Ibaraki University, Ami, Ibaraki 300-0393 Japan; 4 Present address: Koshi Research Station, Institute for Plant Protection, NARO, Kumamoto, 861-1192 Japan

**Keywords:** Entomology, Phylogenetics, Plant hormones

## Abstract

Some herbivorous insects possess the ability to synthesize phytohormones and are considered to use them for manipulating their host plants, but how these insects acquired the ability remains unclear. We investigated endogenous levels of auxin (IAA) and cytokinins (iP and *t*Z), including their ribosides (iPR and *t*ZR), in various terrestrial arthropod taxa. Surprisingly, IAA was detected in all arthropods analysed. In contrast, *t*Z and/or *t*ZR was detected only in some taxa. Endogenous levels of IAA were not significantly different among groups with different feeding habits, but gall inducers possessed significantly higher levels of iPR, *t*Z and *t*ZR. Ancestral state reconstruction of the ability to synthesize *t*Z and *t*ZR revealed that the trait has only been acquired in taxa containing gall inducers. Our results strongly suggest critical role of the cytokinin synthetic ability in the evolution of gall-inducing habit and IAA has some function in arthropods.

## Introduction

Insects are the most speciose taxa in terrestrial ecosystems^1–3^. In terms of feeding habit, almost half of extant insect species are phytophagous, meaning they consume living plant material^4^. Based on the oldest insect fossils and recent molecular studies, insects are estimated to have evolved on the earth at roughly the same time as plants^3,5,6^. It should be noted that phytophagy is uncommon in terrestrial arthropods other than Acari and Insecta. This suggests that the evolution of phytophagy was an important event in the adaptive radiation and diversification of extant insects.

Among phytophagous insects, some taxa have evolved the ability to induce galls on their host plants and can manipulate host plant metabolism and morphogenesis in highly sophisticated ways^7–9^. Phytohormones, particularly auxin (indole-3-acetic acid, IAA) and cytokinins, have been reported to play an important role in gall induction by insects^10–12^. A study has demonstrated that the larvae of a gall-inducing sawfly *Pontania* sp. (Hymenoptera: Tenthredinidae) possess the enzymatic activity necessary to synthesize IAA within their body^10^. In addition, this sawfly probably has the ability to synthesize *trans*-zeatin riboside (*t*ZR), a riboside form of the bioactive cytokinin *trans*-zeatin (*t*Z). The sawfly larvae that exit galls in the autumn possess only low amounts of *t*ZR, whereas the female adults that emerge in spring, after overwintering in the soil, exhibit an extraordinarily high concentration of *t*ZR^10^. Such high concentrations of auxin and cytokinins (or their ribosides) have been detected in some gall-inducing insects, including a cecidomyiid (Diptera)^13^ and a psyllid (Hemiptera)^14^, suggesting that those phytohormones are associated with gall-inducing insects or with galls in various insect taxa.

Surprisingly, the ability to synthesize auxin has been found not only in gall-inducing insects but also in other insects, such as the silkworm, *Bombyx mori* (Lepidoptera), the western honeybee, *Apis mellifera* (Hymenoptera), the common fruit fly, *Drosophila melanogaster* (Diptera), and even in non-phytophagous species including the housefly, *Musca domestica* (Diptera)^15,16^. Most recently, the biosynthetic pathway of IAA was clarified in *B*. *mori* and *Pontania* sp., and some key enzymes involved in the IAA synthesis were identified^17–19^.

On the basis of the above, we hypothesized that insects evolved the ability to synthesize auxin or cytokinin prior to the evolution of phytophagy or gall induction. To explore the evolutionary origins of the ability to synthesize auxin and cytokinins in insects, we comprehensively investigated endogenous levels of these phytohormones in various terrestrial arthropods. We then clarified that endogenous IAA was present in all major groups of terrestrial arthropods, including spiders, mites, crustaceans, millipedes and insects. In contrast, we revealed that only some taxa containing gall inducers seem to have acquired the ability to synthesize *t*Z or *t*ZR in insects. Based on these results, we discuss the possible involvement of the acquisition of an ability to synthesize phytohormones in the evolution of phytophagous and gall-inducing habits in insects.

## Methods

### Preparation of samples and quantification of phytohormones

Various terrestrial arthropods were either sampled from laboratory-reared strains or were collected from the field by direct capture or net-sweeping for flying adults. Detailed collection data are shown in Table S1. During the sampling, we took measures to diminish contamination with plant-derived phytohormones, as follows: living samples (especially herbivores) were starved for two days, or developmental stages not directly associated with living plants (hatchlings, pupae etc.) were used. Each sample consisted of one individual (if they weighed ca. ≥ 5 mg), while for tiny species, several to hundreds of individuals were combined per sample to give 5–10 mg fresh weight (FW). As far as the situation allowed, three replications were prepared for each species. Samples were weighed and frozen as soon as they had been prepared.

The concentration of indole-3-acetic acid (IAA) and cytokinins, such as isopentenyladenine (iP) and *trans*-zeatin (*t*Z), and their ribosides, isopentenyladenosine (iPR) and *trans*-zeatin riboside (*t*ZR), were measured using whole body extracts, according to a previously described method using stable isotope-labelled internal standards^14^.

### Reconstruction of ancestral traits

Ancestral state reconstructions were performed using maximum-likelihood reconstructions analysis and the Markov k-state one-parameter (Mk1) model in Mesquite 3.6^19^. The maximum-likelihood analyses find the ancestral states (the internal nodes) that maximize the probability that the observed character states (the terminal nodes) would evolve under a stochastic model of evolution^21,22^. The Mk1 model assumes that any character change is terminal probable^23^. The tree topology follows the latest phylogenetic tree inferring the relationships among insect orders and their ancestral terrestrial arthropods based on 1478 protein-coding genes^3^. The ability of synthesizing phytohormones in the major taxa of the phylogeny was categorized in the analyses based on our data (Table S2) as follows: (0) absent, (1) present, (2) present and absent. Taxa in which either the bioactive phytohormone or its riboside form was not detected, and the mean concentration of the other form was less than 1.0 ng/g FW were treated as contaminated and were considered lacking the ability to synthesize phytohormones.

### Comparisons of endogenous phytohormone levels among taxa and feeding habits

Endogenous phytohormone concentrations were analysed among taxa (Apterous insects/Paleoptera, Polyneoptera, Condylognatha and Holometabola) and feeding habit (gall inducers, non-galling herbivores feeding on fresh plant parts, and feeding guilds other than herbivores) using generalized linear models (GLM) with a Gaussian distribution. Categorization of feeding habits were based either on literatures^24–26^. The categorization of taxa and feeding habits is summarized in Table S2. Treatment means were compared using Tukey’s HSD test. In cases when interaction effects between taxa and feeding habit were significant, phytohormone concentrations were analysed among feeding habit within each higher taxon (Condylognatha and Holometabola). All statistical analyses were performed using R ver. 4.0.3^27^.

## Results

A certain concentration of IAA was detected in all terrestrial arthropods, including spiders, mites, crustaceans and a millipede, that were analysed in this study (Table S2). The IAA concentrations were extraordinarily high comparing to the concentrations in general plant tissues in some taxa, such as mites, the millipede, an Archaeognatha (bristletail), and a sawfly (> 1000 ng/g FW). Even in the other species, the concentration of IAA was relatively high (237.9 ± 61.8 ng/g FW on average ± SE in all taxa).

The mean concentrations (± SE) of cytokinins were 8.5 ± 3.0, 22.1 ± 4.4, 12.1 ± 3.6 and 7.3 ± 1.9 ng/g FW for iP, iPR, *t*Z and *t*ZR, respectively, which was also relatively high. In contrast to IAA, cytokinins were not detected in some taxa of insects (Table S1). Notably, concentrations of 1.0 ng/g FW or more of *t*Z and *t*ZR were not detected in any taxa belonging to Myriapoda, apterous insects, Paleoptera, or Polyneoptera, although the Polyneoptera include phytophagous orders such as Orthoptera and Phasmatodea. In addition, heteropterans, which also include phytophagous taxa, generally possessed very low levels of *t*Z and *t*ZR. Gall inducers, such as *Gynaikothrips uzeli*, *Tetraneura nigriabdominalis*, *Pontania* sp. and *Rhopalomyia yomogicola*, had relatively high concentrations of *t*Z and *t*ZR. In other arthropods, mites possessed extraordinary high concentrations of iPR (Table S2).

**Figure 1:**
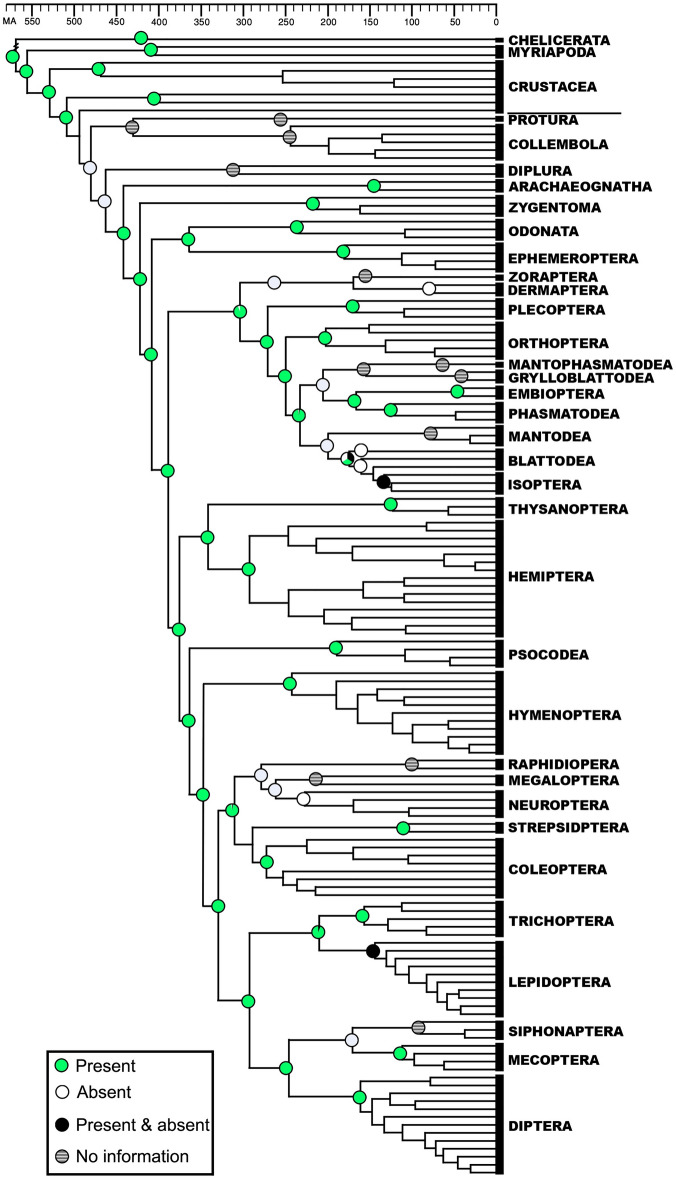
Ancestral state reconstruction of the ability to synthesize isopentenyladenine (iP) and isopentenyladenosine (iPR) in terrestrial arthropods. Circles in nodes represent the percentage of the probability of the reconstructed character state. The time-calibrated phylogenetic tree is modified from (3). Time Scale is in millions of years before present.

**Figure 2:**
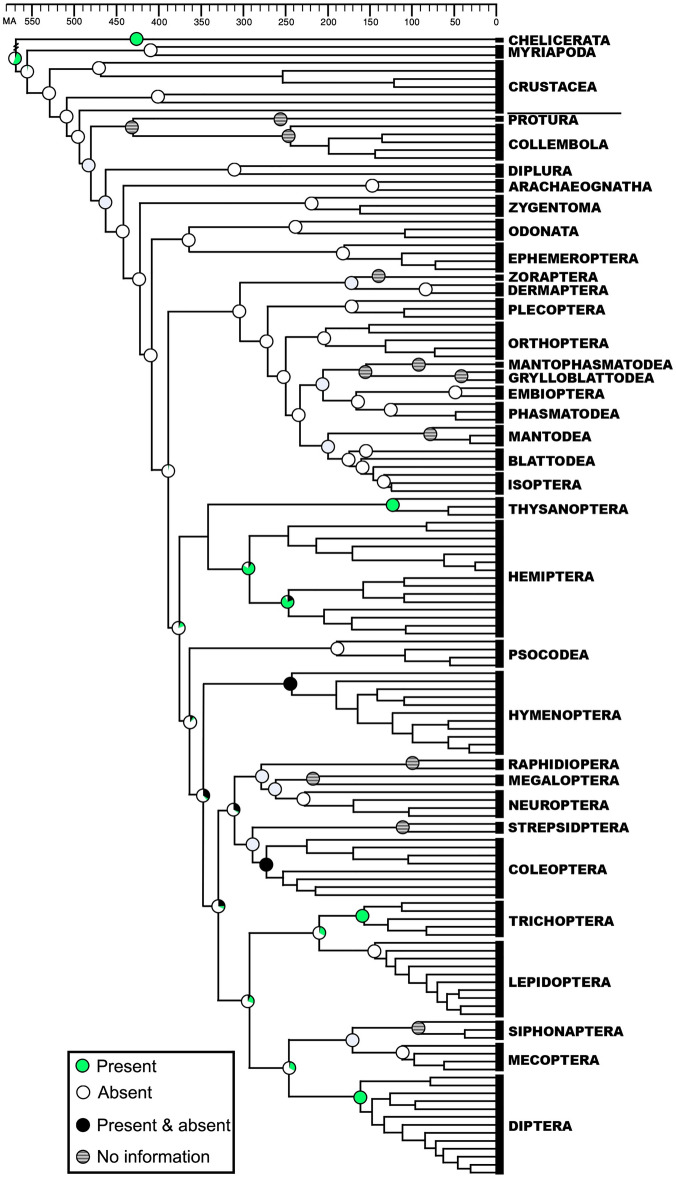
Ancestral state reconstruction of the ability to synthesize *trans*-zeatin (*t*Z) and *trans*-zeatin riboside (*t*ZR) in terrestrial arthropods. Circles in nodes represent the percentage of the probability of the reconstructed character state. The time-calibrated phylogenetic tree is modified from (3). Time Scale is in millions of years before present.

The reconstruction of ancestral traits led us to deduce that inferred common ancestor of insects possessed the ability to synthesize iP (or iPR), but some taxa have since lost this ability (Fig. 1). In contrast, the common ancestor of insects was inferred not to have had the ability to synthesize *t*Z (or *t*ZR), and the ability appears to have been acquired independently in Condylognatha and Holometabola around 300 MA (Fig. 2).

**Figure 3:**
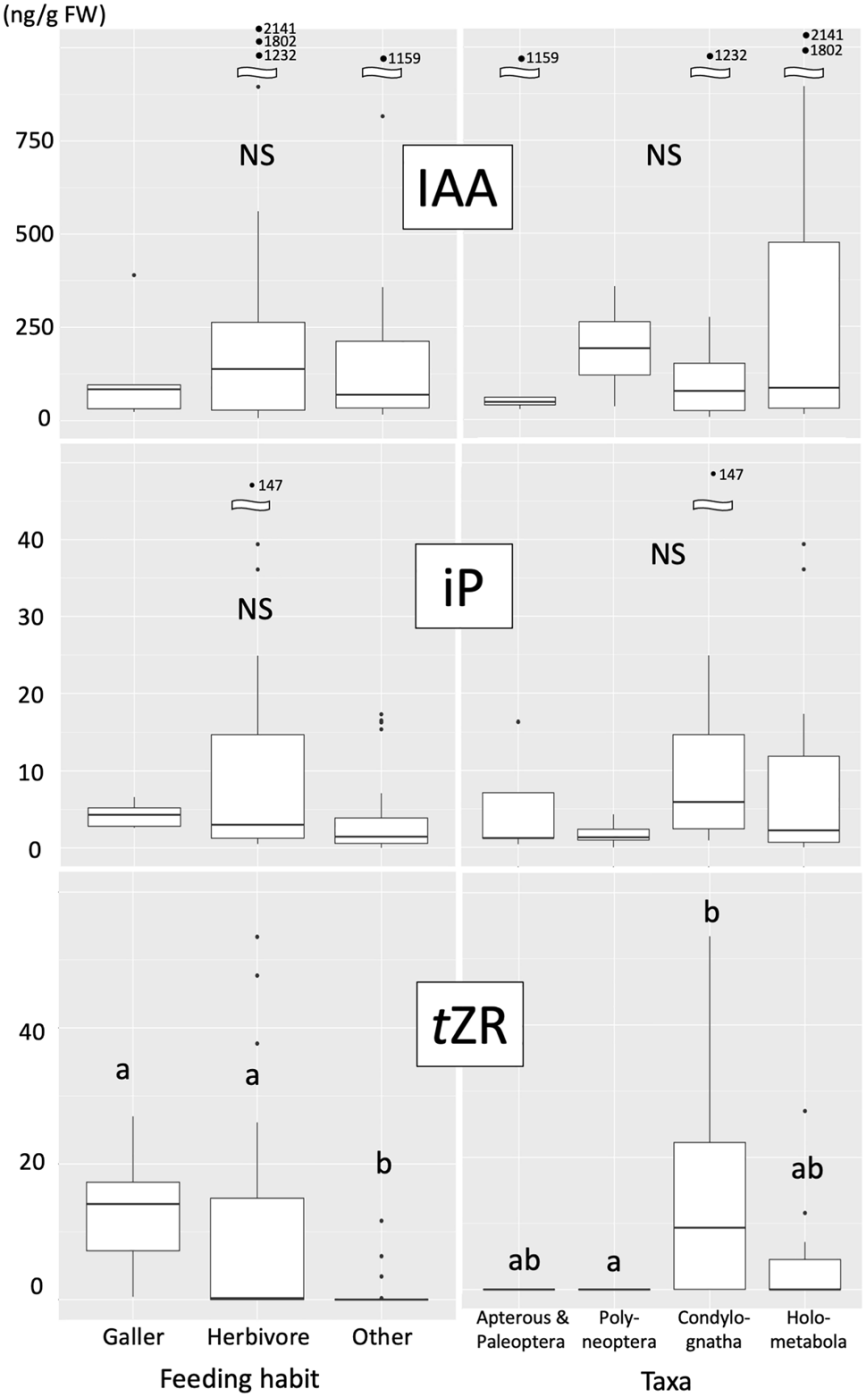
Comparison of endogenous concentrations of auxin (indole-3-acetic acid, IAA), isopentenyladenine (iP), and *trans*-zeatin riboside (*t*ZR) among different taxa and feeding habits (gallers, non-galling herbivores and others) of terrestrial insects. The same letters above bars indicate no significant differences among groups (GLM).

**Figure 4:**
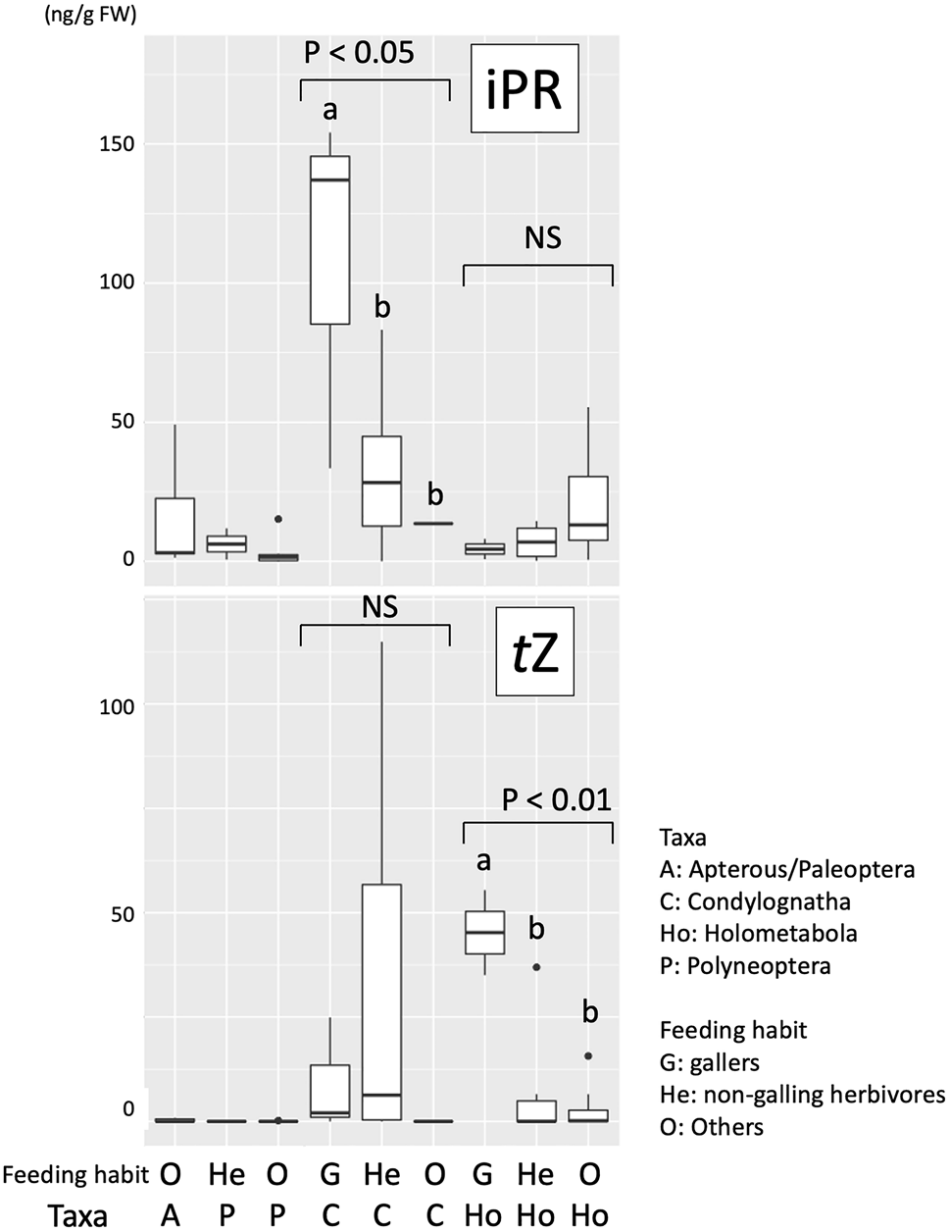
Comparison of endogenous concentrations of *trans*-zeatin (*t*Z), and isopentenyladenosine (iPR) among different feeding habits of insects. Because significant interaction effects between taxa and feeding habit were detected, concentrations were compared among different feeding guilds within Condylognatha and Holometabola, respectively.

In GLM analyses, IAA concentrations were significantly different among feeding habit, but not among taxa (DF = 3, χ^2^ = 7.41, *p* = 0.060 for taxa; DF = 2, χ^2^ = 6.84, *p* = 0.033 for feeding habit; DF = 3, χ^2^ = 4.38; *p* = 0.224 for taxa × feeding habit) (Fig. 3); iP concentrations were not significantly different among both taxa and feeding habit (GLM; DF = 3, χ^2^ = 1.11; *p* = 0.78 for taxa; DF = 2, χ^2^ = 0.95, *p* = 0.621 for feeding habit; DF = 3, χ^2^ = 0.284, *p* = 0.963 for taxa × feeding habit) (Fig. 3); iPR concentrations were significantly different among both taxa and feeding habit and their interaction was also significant (GLM; DF = 3, χ^2^ = 34.46; *p* < 0.001 for taxa; DF = 2, χ^2^ = 52.18, *p* < 0.001 for feeding habit; DF = 3, χ^2^ = 47.712, *p* < 0.001 for taxa × feeding habit) (Fig. 4); in Condylognatha, gall inducers possessed significantly higher concentrations of iPR than non-galling herbivores and others, but no significant differences were detected among different feeding habits in Holometabola (Fig. 4); *t*Z concentrations were significantly different among feeding habit and interaction between taxa and feeding habit was significant (GLM; DF = 3, χ^2^ = 3.39; *p* = 0.336 for taxa; DF = 2, χ^2^ = 16.70, *p* < 0.001 for feeding habit; DF = 3, χ^2^ = 22.65, *p* < 0.001 for taxa × feeding habit) (Fig. 4); in contrast to iPR, no significant differences were detected in *t*Z concentrations among different feeding habits in Condylognatha, but gall inducers possessed significantly higher concentrations of *t*Z than non-galling herbivores and others in Holometabola (Fig. 4); *t*ZR concentrations were not significantly different among both taxa and feeding habit (GLM; DF = 3, χ^2^ = 5.93; *p* = 0.115 for taxa; DF = 2, χ^2^ = 4.60, *p* = 0.100 for feeding habit; DF = 3, χ^2^ = 1.11, *p* = 0.774 for taxa × feeding habit).

## Discussion

In this study, we determined that terrestrial arthropods possess relatively high concentrations of endogenous IAA compared with the concentration of IAA in most plant tissues, i.e., approximately 20 and 24 ng/g FW in *Arabidopisis*^28^ and in *Oryza sativa*^29^, respectively. This phenomenon cannot be explained by sequestration of phytohormones in insect bodies, because not only herbivores but also non-herbivores, even species which do not consume plant materials throughout their life similarly possess certain amounts of phytohormones. In addition, the amounts of phytohormones detected in the insect bodies are two or three orders of magnitude higher than those in ordinal plant tissues. As mentioned earlier, a gall-inducing sawfly (*Pontania* sp.) and some other insect species were experimentally proved to possess the enzymatic activity to synthesize IAA within their body^10^. Although we did not perform an analysis of the ability to synthesize IAA, it is clear that the common ancestor of insects, as well as that of other terrestrial arthropods, possessed endogenous auxin. This suggests the possibility that the auxin synthetic ability has been retained in terrestrial arthropods for a long period. To date, studies of the associations between phytohormones and insects have focused on gall inducers and their host-manipulating mechanisms^12^. In fact, we believe that no researchers have either expected or predicted the presence of phytohormones in such a wide range of terrestrial arthropods, including not only phytophagous insects but also other insect species, in addition to spiders, millipedes and crustaceans. This is surprising and challenges the notion that phytohormones are substances primarily involved in plant signalling. Moreover, we found that the endogenous concentrations of IAA were generally high in terrestrial arthropods, which strongly suggests that IAA and/or enzymes responsible for its synthesis have specific functions in arthropods. In *D*. *melanogaster*, the function of the gene most homologous (Aldox89A) to the aldehyde oxidase of *B*. *mori* is unknown, but the activity of this enzyme increases at pupation and midway through the pupal stage^30^. Comparative studies and functional analyses will be necessary in the future, but we detected IAA in both males and females, as well as in various developmental stages, implying that IAA and/or related enzymes play particular roles throughout the lifecycle stages. Although we did not detect any significant relationships between endogenous IAA levels and feeding habits, at least in some gall inducers IAA is involved in their gall induction^31,32^. For example, fundatrices of the aphid *Tetraneura nigriabdominalis* (Hemiptera), which are responsible for gall induction, appear to actively synthesize IAA during the gall induction initiation process^33^. In our current study, we analysed IAA levels using specimens not directly related to plants, even for herbivores and gall inducers, so the basic level of IAA may be similar among different feeding guilds, and some gall inducers and herbivores may enhance their IAA levels as needed.

The mean concentrations of cytokinins were also somewhat high in comparison with the concentrations of iP, iPR, *t*Z and *t*ZR in most plant tissues: ca. 0.15, 5.0, 0.4 and 5.0 ng/g FW, respectively, in *Arabidopsis*^34^ and 0.18, 0.4, 0.16 and 0.7 ng/g FW, respectively, in *O*. *sativa*^29^. Although a recent study showed the widespread distribution of cytokinins in insects^35^, the investigation was restricted to herbivorous insects belonging to Condylognatha and Holometabola and did not discuss differences between iP- and *t*Z-type cytokinins. Our analysis suggested that the common ancestor of insects also possessed the ability to synthesize iP (or iPR) but, unlike with IAA, this ability seems to have been lost in some taxa. The common ancestor of insects was also thought not to possess the ability to synthesize *t*Z (and *t*ZR). Insects seem to possess genes for tRNA isopentenyltransferase (tRNA-IPT) but not for adenylate-IPT; therefore, they may have acquired the highly effective ability to synthesize cytokinin via prenylation of tRNA. Further studies are needed to clarify the hydroxylation mechanism required to produce *t*Z/*t*ZR. In leaf-mining moths, which induce ‘green island effects’ by the prolongation of cell life in plant tissue surrounding their mines on shed leaves, the endosymbiont *Wolbachia* seems to play a critical role in the production of cytokinins in these insects’ bodies^36,37^. Although information relating to endosymbiotic bacteria in our study is limited, it is estimated that almost 40% of insects possess *Wolbachia* endosymbionts^38^. So, in some insects, endogenous cytokinins may be derived from *Wolbachia* or other symbionts. However, a recent study suggested that specific bacterial symbionts are not involved in gall induction by insects^39^. Moreover, in the gall-inducing sawfly *Pontania* sp., which contains very high quantities of *t*Z and *t*ZR^10^, studies involving both de novo RNA-seq using rRNA-depleted mRNA and de novo draft genome sequencing detected only a single insect-derived IPT gene (Suzuki et al., unpublished data). So, these insects are likely to have the ability to synthesize cytokinins without any assistance from symbionts.

In cytokinins, we found that gall inducers and non-galling herbivores possessed higher concentrations of *t*ZR than other feeding guilds. These results imply that herbivorous insects may use cytokinins when they feed on their host plants for some purposes (e.g., disturbance of antiherbivore defense). A recent study demonstrated that saliva of the fall armyworm *Spodoptera frugiperda* contains some phytohormones, such as jasmonic acid, salicylic acid and abscisic acid, and non-protein components^40^. These phytohormones are suggested to modulate plant defensive responses^40^. Furthermore, gallers possessed higher concentrations of iPR in Condylognatha and *t*Z in Holometabola. In addition, major phytophagous orders, including Orthoptera, Phasmatodea and Heteroptera, largely lack endogenous *t*Z and *t*ZR and, notably, the gall-inducing habit has not evolved in these taxa^41^. These imply that phytohormones mainly used by Condylognatha and Holometabola are somewhat different from each other. Moreover, the acquisition of the ability to synthesize *t*Z seem to have played a critical role in the evolution of the gall-inducing habit in Holometabola. Gall inducers are well known to use these phytohormones in manipulating plant tissue^10,12^. In *Arabidopsis*, *t*Z-type cytokinins are distributed mainly in xylem sap and transported from the roots to the aboveground parts, while iP-type cytokinins are distributed in phloem sap and transported in the opposite direction^42^. In crown gall formation caused by *Agrobacterium*, *t*Z-type cytokinins were shown to be more effective at gall induction than iP-type cytokinins^43,44^, possibly because of the lower affinity of *t*Z than that of iP for cytokinin oxidase, an enzyme responsible for the degradation of cytokinins^43^. In our previous study using a gall-inducing leafhopper, *Cicadulina bipunctata*, and susceptible and resistant varieties of maize, a significant increase in *t*Z (and not IAA and iP) was detected only in the susceptible variety of maize on which galls were induced^11^. The *t*Z concentrations are slightly high in gallers and non-galling herbivores belonging to Condylognatha and this is contrastive to the fundamental lack of this phytohormones in other feeding guilds as well as in Polyneoptera, Paleoptera, and apterous insects. Further studies are needed to clarify the importance of *t*Z type cytokinin in gall induction by insects. High concentrations of cytokinins were also detected in mites, which also include herbivores and gall inducers. The cytokinin synthetic ability of mites and its involvement of phytophagy and gall induction is an interesting future study subject.

In conclusion, in this study we showed that a broad range of terrestrial arthropods possess relatively high concentrations of IAA within their bodies. It is suggested that the common ancestor of insects possessed the ability to synthesize IAA and iP (or iPR). In contrast, the ability to synthesize *t*Z and/or *t*ZR was inferred to have been acquired in just a few insect taxa, including gall inducers. Specifically, gall inducers possessed a higher concentration of some cytokinins than other feeding guilds, a strong indication of the importance and involvement of these phytohormones in the evolution of gall-inducing habits among terrestrial arthropods.

## Supplementary Information

Supplementary Information.
