# Supplementary material for: Terrestrial arthropods broadly possess endogenous phytohormones auxin and cytokinins
Source: Sci Rep. 2022 Mar 19;12:4750. doi: 10.1038/s41598-022-08558-6 (PMC8934337; doi:10.1038/s41598-022-08558-6)
Supplement: Supplementary file 1 — Supplementary Information. [file 41598_2022_8558_MOESM1_ESM.docx]

Legends for supplementary tables

Table S1. Data collected and categorization of terrestrial arthropods used in the analysis.

Table S2. Endogenous concentrations (ng/g FW) of auxin (IAA) and cytokinins (iP, iPR, tZ and tZR) in terrestrial arthropods.

Table S1. Data collected and categorization of terrestrial arthropods used in the analysis.

Table S2. Endogenous concentrations (ng/g FW) of auxin (IAA) and cytokinins (iP, iPR, tZ and tZR) in terrestrial arthropods.
